# Balancing Automatic-Controlled Behaviors and Emotional-Salience States: A Dynamic Executive Functioning Hypothesis

**DOI:** 10.3389/fpsyg.2016.02067

**Published:** 2017-01-19

**Authors:** Bruno Kluwe-Schiavon, Thiago W. Viola, Breno Sanvicente-Vieira, Leandro F. Malloy-Diniz, Rodrigo Grassi-Oliveira

**Affiliations:** ^1^Experimentelle und Klinische Pharmakopsychologie, Psychiatrische Universitätsklinik ZürichZürich, Switzerland; ^2^Developmental Cognitive Neuroscience Lab, Graduate Program in Psychology, Pontifical Catholic University of Rio Grande do Sul (PUCRS), Porto AlegreBrazil; ^3^Developmental Cognitive Neuroscience Lab, Graduate Program in Pediatrics and Child Health, Pontifical Catholic University of Rio Grande do Sul (PUCRS), Porto AlegreBrazil; ^4^Department of Mental Health, School of Medicine, Federal University of Minas GeraisBelo Horizonte, Brazil; ^5^LUMINA Neurosciences and Mental Health InstituteBelo Horizonte, Brazil

**Keywords:** executive functions, cognitive control, self-regulation, neuropsychology, automatic process, reasoning, stress, psychological

## Abstract

Recently, there has been growing interest in understanding how executive functions are conceptualized in psychopathology. Since several models have been proposed, the major issue lies within the definition of executive functioning itself. Theoretical discussions have emerged, narrowing the boundaries between “hot” and “cold” executive functions or between self-regulation and cognitive control. Nevertheless, the definition of executive functions is far from a consensual proposition and it has been suggested that these models might be outdated. Current efforts indicate that human behavior and cognition are by-products of many brain systems operating and interacting at different levels, and therefore, it is very simplistic to assume a dualistic perspective of information processing. Based upon an adaptive perspective, we discuss how executive functions could emerge from the ability to solve immediate problems and to generalize successful strategies, as well as from the ability to synthesize and to classify environmental information in order to predict context and future. We present an executive functioning perspective that emerges from the dynamic balance between automatic-controlled behaviors and an emotional-salience state. According to our perspective, the adaptive role of executive functioning is to automatize efficient solutions simultaneously with cognitive demand, enabling individuals to engage such processes with increasingly complex problems. Understanding executive functioning as a mediator of stress and cognitive engagement not only fosters discussions concerning individual differences, but also offers an important paradigm to understand executive functioning as a continuum process rather than a categorical and multicomponent structure.

## Introduction

The conceptualizations of executive functions have emerged from the observation of patients who had suffered frontal lobe lesions and became unable to manipulate, integrate, and respond to internal and external stimulus in the same way they used to do (Jurado and Rosselli, 2007; Goldstein et al., 2014). Throughout the last few decades, a range of models have emerged and executive functions has become a multifaceted mental concept that includes more than 30 different components (Barkley, 2001, 2012). Although these components were assumed to be interrelated, their exact relationship has not been clearly elucidated. After more than 40 years of studies, there is no consensus regarding the definition of executive functions. The concept previously included a variety of behaviors that were broadly accepted as “high-order cognitive processes,” such as inhibitory control, attention shifting, working memory, goal-directed behavior, and strategic planning [for an extended review of definitions please see (Goldstein et al., 2014)].

Based upon recent neuroscientific findings and theories of cognitive sciences, this article questions this hierarchical characteristic, as well as the multicomponent categorical approaches, commonly attributed to executive functions, by presenting a novel and testable dynamic executive functioning hypothesis. Consequently, different from the majority of reviews on this topic (Miyake et al., 2000; Collette et al., 2005; Jurado and Rosselli, 2007; Tirapu-Ustarroz et al., 2008a,b; Kluwe-Schiavon et al., 2012; Goldstein et al., 2014), it is not the aim of this article to perform an updated overview concerning the theoretical models of executive functions, nor discuss current evidences for the reliability of unitary or multiple components models, and we do not aim to describe the subcomponents that were mostly accepted as “executive”.

In order to accomplish our goal, the article is organized into three sections. In the first section, we briefly discuss the hierarchical and categorical framework that supports the majority of the models of executive functions, especially dual-processing models. In the second section, we highlight a selection of neuroscientific evidences to discuss stress as a core factor behind executive functioning phylogeny, particularly, that stress should be included in executive functioning models as a continuous variable leading to different levels of homeostasis disturbance and, as a consequence, cognitive engagement. The third section introduces the executive functioning hypothesis, elucidating similarities and differences between this perspective and some of the current theoretical models in the field. In this section, we also discuss that the so-called “executive” behaviors (e.g., set-shifting, cognitive flexibility, inhibition, and updating) that could be comprehended as consequences of a permanent adaptive switching between reflexive, conditioned, and goal-oriented behaviors, instead of core individual, but interrelated, cognitive components. Finally, the fourth section of the article discusses subsequent testing of our executive functioning hypothesis and future perspectives.

## The Hierarchical Framework of Executive Functions

In an extensive review regarding executive functions, Goldstein et al. (2014) clarified that the first hypotheses concerning the role of prefrontal cortex (PFC) in human cognition were based upon the theoretical backgrounds of selective attention and multi-store memory models. These models suggested different linear schemas, such as the Bottleneck theory of attention from Donald Broadbent or the three component model from Richard Atkinson and Richard Shiffrin, in order to explain how environmental information are perceived, buffered, and retrieved for conscious awareness (Atkinson and Shiffrin, 1971; Goldstein et al., 2014). Although these models were able to distinguish automatic and controlled cognitive processes, they did not completely explain how information could be deliberatively selected or inhibited during demanding attentional tasks. To fill this gap, the term “cognitive control” was introduced in Posner and Snyder (1975) to describe the capacity to manage thoughts and emotions, allowing people to adapt behaviors across situations according their goals (Goldstein et al., 2014).

Together with previous studies from Alexander Luria, these dual-processing models – and subsequent models, such as the Supervisory Attentional System (SAS) from Norman and Shallice (1983) – were crucial to including the PFC as the main brain structure involved in cognitive control, which was capable of managing and regulating automatic behaviors (Luria, 1970; Norman and Shallice, 1983).

The differences between automatic and controlled processes driven by the cognitive revolution in psychology [for a review, please see (Miller, 2003)] favored a hierarchical and categorical approach, in which cognitive processes were organized as independent, but inter-related components according to their main functions, and aimed to decode the information processing pathway between a stimulus and behavioral response. The multicomponent working memory model proposed by Alan Baddeley and Graham Hitch (Baddeley, 2012) could be viewed as an example of this hierarchical and categorical reasoning, in which three distinct slave systems are coordinated by a central executive. However, according to Baddeley (2012), even though the central executive is the most complex component of working memory, it could be seen as a homunculus that represents a marker of issues requiring explanation. Although Baddeley (2012) suggested that in due course the homunculus might be pensioned off, several models remain convinced that the PFC is the “final frontier of neuropsychology” at the “center of human nature” (Stuss, 2011), and that executive functions “are at the heart of all socially useful, personally, enhancing, constructive, and creative activities” (Lezak, 1982). In other words, hierarchical and categorical approaches have often referred to executive functions as a homunculus that inhibits our instincts and guides our rational behavior.

This categorical approach can be widely observed in the executive functions multicomponent models. Examples include the four-component model hypothesized by (Lezak, 1995), as well as the three core functions summarized by Diamond (2013). The four-component model suggests that executive functions consist of those capacities that enable a person to engage successfully in independent, purposive, and self-serving behavior (Lezak et al., 2012), such as volition, planning, purposeful action, and effective performance (Goldstein et al., 2014). Diamond relies upon the assumption that there is a general agreement regarding three core executive functions: behavioral/cognitive inhibition (including selective attention), working memory, and cognitive flexibility (Diamond, 2013). These functions encourage individuals to not act impulsively, hold information to solve problems, and apply different approaches to a problem when facing new rules or priorities (Diamond, 2013).

In general, both models were based upon clinical experience and observation and they became expressive frameworks in the neuropsychological field. According to our perspective, the three main contributions of these models are: (i) hierarchical and multicomponent approaches make it easier to define some behaviors that seem to represent cognitive processes that could not be classified as general automatic responses caused by a stimulus (e.g., planning and cognitive flexibility); (ii) the possibility to develop specific tasks to assess each component independently (even theoretically considering that they are inter-related), which fits with one of the main aims of neuropsychology as a clinical field, to assess and treat patients with brain injury or disease; and (iii) the possibility to provide an explanation as to how executive dysfunction affects all aspects of behavior differently from specific cognitive deficits (Lezak et al., 2012). However, multicomponent models of executive functions are based upon the traditional framework of “cognitive control” proposed by Posner and Snyder (2004), in which the PFC plays an “executive” role over goal-oriented behaviors (Pribram, 1973) and emotional self-regulation (for a review on this topic please see (Peterson and Welsh, 2014). Medical imaging technologies, developmental research, experimental psychology, and neurosciences have rescued dual-processes theories to describe the so-called “cold” and “hot” cognitive information processing systems (Sahlin et al., 2010; Zelazo and Carlson, 2012).

Once more, many dualistic models have been proposed to characterize these systems for an extensive review see (Evans, 2008). Usually, System 1 (or Type 1) demands stronger activation of subcortical structures and could be defined as unconscious, rapid, automatic, and allowed parallel information processing; while System 2 (or Type 2) demands stronger activation of cortical structures and could be defined as conscious, slow, deliberative, and mostly responsible for serial information processing (Kahneman, 2011; Noël et al., 2013). The idea of an “emotional versus rational” thinking or neural system has found support in several studies and has been extensively used to describe cognitive changes associated with psychiatric disorders and/or neurodevelopment. Some inhibition dysfunction theories of addiction, for example, suggest that chronic drug use reduces self-control, which is needed to inhibit the hedonic impulse to take the rewarding drug again [for a review about models of addiction please see (Emcdda, 2014)]. According to the dual-processing framework of addiction, the neuroplasticity induced by addictive drugs triggered by epigenetic mechanisms impacts proteins in an intracellular level, modifying neurotransmitter signaling in various neuronal circuits leading to an imbalance between those areas that are associated with emotions and reward (e.g., orbitofrontal cortex, ventral striatum, and the limbic system) – usually recruited during situations with stronger affective salience (e.g., facing conditioned drug cues or stress) – and those areas that are associated with more purely cognitive processing and the activation of the dorsolateral parts of the PFC (Volkow and Baler, 2014). Moreover, the imbalance between “hot” and “cold” brain systems has also been used to describe and explain risk-taking behaviors during adolescence, such as unprotected sex, criminal behavior, drug use and abuse, and accidents (Gladwin et al., 2011). Beyond social and environmental factors, neurodevelopmental researchers emphasized that the relatively early maturation of the “hot” affective-motivational bottom-up system and the more slowly developing “cold” top-down control system could explain impulsive behaviors due to the difficulty in delaying gratification (Benningfield et al., 2014), weighing of risks and benefits of a set of actions (Pripfl et al., 2013), and use of ongoing outcomes of these actions to monitor their own performance (Kluwe-Schiavon et al., 2016).

Although dual-process models have explained important issues, especially regarding decision-making, there are studies suggesting that these models might be outdated (Reyna and Brainerd, 2011; Gladwin and Figner, 2014). First, some authors argue that dual-processing models cannot supply and predict mechanisms for developmental reversals in cognition during development, such as increased reasoning biases from childhood to adulthood (Kahneman and Tversky, 2000; Reyna and Brainerd, 2011). Second, the boundaries between “hot” and “cold” executive functions are not clear and, considering previous theories regarding cognitive automaticity (Bargh, 1992; Moors and De Houwer, 2006). Consequently, individual factors (e.g., personality traits or mood), developmental factors (e.g., age and life experiences) and/or contextual factors (e.g., healthy or financial decisions) – could influence the “warmth” of a task (Peterson and Welsh, 2014), thus requiring research to use decompositional approaches (Moors and De Houwer, 2006). In fact, it is true that earlier dual-processing models argued that a process is neither fully controlled nor automatic (Bargh, 1992). Dualistic conceptions (as well as the central executive homunculus of Baddeley) were thought to be didactically used to describe and explain complex behaviors. However, the exception has become the rule and these dualistic conceptions are replicated and measured as two independent categories instead of two poles of the same gradient. In other words, it is acceptable that “cold” executive functions are measured with tasks that demand planning, working memory, and concept formation, while “hot” executive functions are measured with tasks that demand social cognition, empathy, and emotion regulation (Zimmerman et al., 2016). In the next section we briefly highlight a selection of studies that support the idea that an executive functioning model should be thought of as a frontal-subcortical circuit, in which emotions (here stress) directly modulate the cognitive processes and vice-versa.

## Neuroscientific Findings Toward Executive Functioning

The idea that executive functions are not exclusive related to frontal-cortical areas, but would involve frontal–subcortical neuronal circuits is not new (Leh et al., 2010). Based upon an evolutionary perspective, Ardila (2008) emphasized that executive functions are mediated by dynamic and flexible neuronal networks, questioning the central role of PFC in the executive functions and, afterward, discussing how the executive functions may have evolved in our species. Nonetheless, questioning the central role of PFC in relation to executive functions goes beyond suggesting that executive functions involve subcortical networks, but in fact this also allow us to question the hierarchical perspective in which PFC exerts control over impulses and should be taken as the center of rationality. Note that we do not intend to argue here that the PFC does not exert a key role in cognitive and response inhibition, but that there are enough evidences suggesting that the relationship between cognition and emotion could be more complex than the old-fashioned reasoning that the first (e.g., central executive, superego, or PFC) should control the second (e.g., impulses, id, or limbic system). In this sense, here we focus on those studies some level of stress is necessary to motivate the organism to act and allocate cognitive resources to controlled processes, such as problem solving, monitoring, and updating. After a certain level of stress, the cognitive resources are reallocated in favor of more automatic processes, decreasing working memory span and, in the last instance, increasing response inhibition and unconditioned behaviors, such as fight or flight responses. Additionally, we mainly focus upon acute stress research since in the biological and psychological fields the term has been commonly used to describe external events capable of disrupting organism stability or homeostasis.

The executive functioning hypothesis is supported by studies that suggest that the PFC, especially medial areas, coordinates the brain circuits that mediate emotional responses (Hermans et al., 2014; McKlveen et al., 2015). This idea was deeply investigated under the somatic marker hypothesis, which demonstrated that the ventromedial PFC and its projections to the orbitofrontal cortex are involved in both emotional response and cognition (Bar-On et al., 2003; Li et al., 2010). The somatic marker hypothesis suggested that body signals (somatic markers) are represented and regulated in the ventromedial PFC, since these somatic markers were not found in people with lesions in this area, which are also correlated with poorer performance on decision-making tasks (Dunn et al., 2006). Additionally, the orbitofrontal cortex is known to show an increased activity in response to stress and it also is implicated in many cognitive functions, such as working memory. In this sense, pre-clinical research has that acute stress can enhance working memory performance by selectively increasing glutamatergic signaling in PFC pyramidal neurons (Yuen et al., 2009). However, the extent to which stress can have a positive or negative effect on specific cognitive functions remains unclear. Barsegyan et al. (2010), for example, demonstrated that acute stress triggers working memory impairment and concurrent enhancement of memory consolidation. Interesting, the authors emphasized the interaction of glucocorticoid receptors and catecholaminergic activity, suggesting that working memory impairment and enhancement of memory consolidation shared a common neural influence within the medial PFC via a common activation of the noradrenergic signaling pathway (Barsegyan et al., 2010).

These data reinforce the idea that the medial PFC is an important integrator of the neuroendocrine and autonomic systems, acting as a coordinator of stress responses. In addition, these data also indicate that stress can be considered as a stimulus that allocates energetic systems to respond to an ongoing or anticipated challenge. McKlveen et al. (2015) discussed that this allocation of energetic systems may occur mostly via hypothalamic-pituitary-adrenal axis (HPA-axis) activation that culminate in the release of glucocorticoids and catecholamines, which in turn leads to several alterations in different brain systems in order to promote adaptive behaviors. As an example of allocation of energy systems, Hermans et al. (2014) have shown that acute stress shifts the phasic activation of locus coeruleus toward a tonic mode of activity, guiding attentional resources for potentially salient information. This allocation of energy resources could explain different behavior patterns observed during stress.

In this regard, two meta-analyses were performed to investigate the effects of psychosocial stress on executive functions (Shields et al., 2016) and the effects of stress on decisions made under uncertainty (Starcke and Brand, 2016). In the first, the authors found that acute stress impaired working memory and cognitive flexibility in humans. Furthermore, Shields et al. (2016) suggested that within inhibition, stress impaired cognitive inhibition (selectively attending to or ignoring information) but enhanced response inhibition. Concerning decision-making, Starcke and Brand (2016) found that stress had significant effects only in those situations in which increased reward seeking and risk taking is disadvantageous and discussed that this finding could be explained by two mechanisms: the first suggests that acute stress should increase the reliance on immediate and high rewards via alterations in dopamine release at the cost of considering potential losses; while the second mechanism suggests that stress may lead to unsystematic decisions without considering all of the options and may generally impair executive control via reductions of prefrontal functioning (Shields et al., 2016). Taken together, their findings are in agreement with the current perspective that stress reallocates limited executive resources in adaptive ways to facilitates adaptive decisions, although the authors highlight that it is not clear what executive function receives these reallocated resources and why (Shields et al., 2016).

It is also hypothesized that stress-induced shifts in cognitive functions occur because two different large-scale neuronal networks (salience network and executive control network) may compete for limited resources, and as proposed by Hermans et al. (2014), regulate externally directed attention. This model goes further than the majority of “hot” and “cold” dualistic perspectives because it introduces a dynamic interaction between the delay between stressor onset and task performance, and also because it considers the prefrontal areas as the key structures supporting this “competition.” In a second meta-analysis, Shields et al. (2015) investigated the effects of acute cortisol administration on executive functions, focusing upon working memory inhibition and set-shifting shifting. After separating the genomic effects of cortisol (slow-acting effects caused by the modulation of gene expression) from its non-genomic effects (rapid-acting effects without the modulation of gene expression) by controlling for the delay between cortisol administration and cognitive testing, the authors found interesting and divergent effects of the hormone in cognition according to different time-windows post-administration. The authors suggested that the non-genomic effects of cortisol significantly impair working memory between 15 and 73 min post-administration, but begins to improve working memory after this period. However, the same effects of cortisol improve inhibition from 15 to 135 min post-administration, but begins to impair inhibition after this period, and no effects where found related to set-shifting (Shields et al., 2015). This data is in accordance with the idea that stress levels modulate the allocation of cognitive resources in a dynamic perspective, increasing inhibitory control and decreasing working memory capacity, which we hypothesized could facilitate the organism to engage in a logic deliberative reasoning to solve the problem. Nevertheless, if this strategy was not sufficient to solve the problem, individuals might use the working memory as an automatic adaptive cognitive mechanism to guide behavior, demanding less logic deliberative reasoning. Moreover, this meta-analysis emphasized that the time course difference between salience network and executive control network to reach the peak and then return to the baseline could also be an important feature to comprehend different effects of stress in the executive functions. In the acute phase, neural resources are allocated toward the salience network and the executive control network is actively suppressed, while in the recovery phase, this effect is reversed.

Here we argue that, at a primary level, a minimum amount of stress is required in order to motivate the organism to act and cognitively engage in problem solving by decreasing working memory capacity. Unfortunately, there are few studies directly investigating these effects and the majority of evidences in this direction are based upon clinical studies suggesting that stress could decrease the threshold to act. For example, some authors suggest that schizophrenia is primarily a frontostriatal disorder (Liu et al., 2011), in which executive functions deficits and deterioration are a central aspect of the disease [for a review see (Kluwe-Schiavon et al., 2013)]. In this sense, recent findings in schizophrenia have reconceptualised context processing as a function of proactive and reactive cognitive control (Aron, 2011). Proactive control can be comprehended as a form of default mode activated by goal-relevant information before the occurrence of cognitively demanding events, to optimally bias attention, perception, and action systems in a goal-driven manner; while reactive control is recruited only after the detection of a high-interference event, favoring attentional control and response inhibition (Anticevic et al., 2013). The proactive control depends upon the updating and maintenance of contextual information, which in turn, are associated with Gamma-aminobutyric Acid (GABA) and glutamate neurotransmitter mechanisms and *N*-methyl-_D_-aspartate (NMDA) receptor functioning. There are evidences suggesting that in schizophrenia, the connectivity between dorsolateral PFC and other cognitive control related brain regions are associated with dopamine and GABAergic signaling impairments, which support the information representation in dorsolateral PFC (Barch and Ceaser, 2012). Thus, such dysfunction in dopamine and GABAergic signaling may explain, in part, some of the behavior difficulties observed in schizophrenia related to proactive control, such as engaging in a conversation or planning. The notion of proactive control fits an executive functioning perspective because when facing a minimum level of stress, patients diagnosed with schizophrenia may have difficulties in allocating executive functioning resources necessary to properly engage in adaptive behavior.

Therefore, it seems unlikely that a “hot” vs. “cold” dichotomy will remain as a source of hypotheses for research in cognitive and experimental neuroscience. Although these dual-processes models have contributed to the understanding of information processing and brain disorders (Volkow and Baler, 2014), research questions centerd upon a categorical epistemological base seem to defy recent findings in the field (Morris and Cuthbert, 2012). Current interdisciplinary efforts to integrate “hot” and “cold” processes is timely and important since psychological scientists have previously assumed that adaptive behavior in real-world contexts involves continuous interactions between emotional and cognitive processes (Peterson and Welsh, 2014).

Taken together, our main goal here is to highlight that although the literature converges toward a dynamic role of PFC as a coordinator of stress adaptation, executive functions literature appears to be mostly focused upon hierarchical and categorical approaches. More than inter-related components that exert control over emotional salience, we propose that executive functioning should be comprehended as the main processes behind the allocation of cognitive resources in the face of a challenge. In this sense, instead of looking for energization, one can investigate the amount of stress that is needed to motivate the organism to act; instead of looking for problem solving or inhibitory control, one can investigate how long the organism can keep the executive control network engaged without shifting energy resources for working memory and salience network during a mild, moderate, or severe stress challenge. Instead of looking for cognitive flexibility, one can investigate how fast the organism can retrieve and adapt conditioned behavior schemas to cope with a new scenario. This dynamic perspective considers executive functioning as continuum process that could be used to identify how adaptable the organism is to an unpredictable environment.

## A Dynamic Executive Functioning Hypothesis

Our dynamic executive functioning hypothesis emerged from a fundamental question raised by Barkley (2001), “Why did humans develop executive functions?” Taking an evolutionary perspective, we argue that executive functioning emerged from: (a) the ability to solve immediate problems and generalize successful strategies; and (b) the ability to synthesize and organize environmental information in view of identifying uniformities that allow predictions about nature and future. From our perspective, these abilities are intrinsically related with two fundamental assumptions that are omitted by the majority of executive functions models. The first is a motivational feature that refers to the necessity of an existent problem to be solved, in other words, a motivational variable that might stress the organism and instigate the goal-oriented behavior. The second refers to the optimization of future solutions based upon previous experience, which is an ontogenetic assumption based upon the combination of the ability to generalize successful strategies along development and the ability to predict problems. The first assumption instigates the necessity to include a motivational variable derived from an internal or external stressful event capable of disturbing the organism homeostasis. Thus, this motivational aspect should be comprehended as a that represents levels of homeostasis disturbance. The second assumption suggests that executive functions could be considered a “cyclical” process (that is why we refer to it as executive “functioning”), in which the main goal is to automatize efficient solutions that were cognitively demanding in the past, enabling individuals to allocate cognitive resources to the executive control network to solve new complex problems.

**FIGURE 1:**
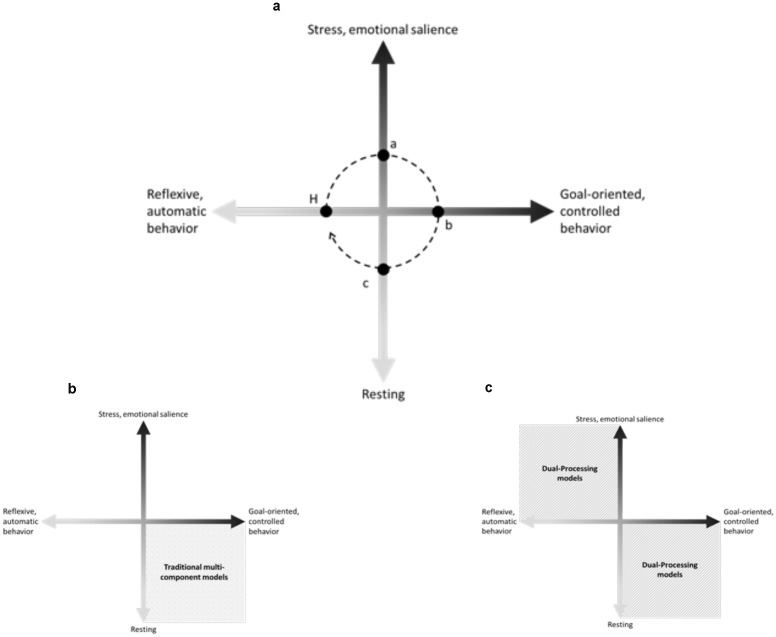
**Dynamic executive functioning hypothesis. (A)** Dynamic executive functioning hypothesis: our theoretical hypothesis suggests that organisms tend to maintain a homeostatic state (H). Stress occurs, when any environmental demand gradually triggers goal-oriented behaviors, firstly using habitual responses and adapting it to adjust to the new demand. In an optimal executive functioning, when the ongoing behavior is insufficient to respond to environmental demands the emotional salience network reaches a peak (a), the organism would be able to inhibit disruptive automatic behaviors such as fight or flight responses and to use the available information (emotional salience and previous behaviors) to successfully respond to the environmental demand, decreasing the emotional salience and decoding the new contingencies (b). Once the new contingencies are decoded, the organism would repeatedly use the successful behavior (c) to completely solve the environmental demand and, then, returning to the homeostatic state (H). **(B)** Traditional multicomponent models. **(C)** Dual-processing models.

Here we proposed a schematic model to illustrate that the executive functioning could be comprehended as a balance between the salient network and the executive control network (**Figure 1a**). Differently from the majority of dual-processing models in which the strongest activation of salient network necessarily culminates in the weakness of cognitive executive control network, our theoretical hypothesis suggests that the strength and the direction of the relationship between these two networks would be indicative of optimal or impaired executive functioning. In optimal executive functioning, the organism should be able to quickly adjust the ongoing behavior when faced with a stressful event. To do so, the organism should retrieve previous successful strategies and should be flexible enough to adapt these strategies if necessary. The executive functioning lies in the amount of stress necessary to motivate problem solving and the amount of cognitive effort demanded to solve the problem. If the organism is not sufficiently sensitive to identify potentially stressful events and to initiate an adaptive response in time, this would suggest a failure in updating environmental information. If the problem-solving demands a greater amount of cognitive effort that does not correspond to the difficulty level of the task, this results in a failure in monitoring the ongoing behavior or in a lack of cognitive flexibility. If the organism is not able to maintain the ongoing goal-oriented behavior with a certain level of stress, or if the organism shifts to habitual (or reflexive) responses, even with a minor increase of the stress levels, this would suggest a failure in inhibitory control.

In this perspective, traditional executive components (i.e., updating, monitoring, problem solving, cognitive flexibility, and control inhibition) could not be accessed without taking into account their role in the entire adaptive process and the optimization of cognitive effort due to a perceived stress. In this sense, different from the majority of traditional multi-component models of executive functions that emphasized high-order quality of the cognitive processes, or the majority of dual-processing models that expanded the traditional models investigating the “mirrored” features (**Figures 1b,c**), the dynamic executive functioning hypothesis – based upon the salience and executive control neuronal networks proposed by Hermans et al. (2014) – intended to set aside these conceptual categorical borders commonly used to define executive behaviors, suggesting that an optimal adaptive process would consider the constant interaction between an emotional salience axis and executive controlled axis.

As shown in **Figure 1**, executive functioning would: (1) constantly monitor the environment, adjusting the ongoing behavior as soon as new demands are identified; and (2) promote the automation of successful behavioral strategies. The first point is in accordance with the first assumption presented in the beginning of this section. Considering that executive functioning is an adaptive process, it means that without any environmental demand the organism remain in a homeostatic state, for example the so-called Default Mode Network that usually is found when neuroimaging studies investigate participants at rest (Damoiseaux et al., 2006). However, even without a demand capable of triggering goal-oriented behaviors, people still need to monitor and perceive environmental changes, which is likely supported by the synchronicity found between the brain salience network and default mode network at rest, whose disruption falls over dysfunctional thinking (Orliac et al., 2013). The second point means that as soon as a new goal-oriented behavior achieves success in responding to environmental demand, it should be added to the repertoire of successful behavioral strategies of the organisms. This point refers directly to the second assumption also presented in the beginning of this section, which claims that executive functioning should be comprehended as a continuous adaptive process that enables the organism to save cognitive resources when faced with the same environmental demand. This aspect has a key evolutionary purpose since the organism would be able to retrieve previous information to solve similar environmental demands without allocating additional efforts to learn completely new strategies. Even though automaticity has been largely discussed and still lacks consensus, most views of automaticity share the assumption that training and repetition may lead to changes in effort and cognitive demands (Moors and De Houwer, 2006).

Furthermore, executive functioning could be exemplified in daily life situations, particularly, since when we face a new environmental demand (an important appointment or any unpredictable event) cognitive resources should be allocated to cope with that event. Indeed, even relatively low-level cognitive processes can be regulated through environmental stimulus, such as priming. Some authors have suggested that selective attention could be comprehended as a strategic self-regulatory process, since it allows that individuals focus their attention on the goal-relevant information (Fitzsimons and Bargh, 2004). Thus, it seems to be counterintuitive to think that an individual can be emotionally engaged with a certain task while no cognitive resources are allocated to cope with it, at least, on some basic attentional level.

To give another example of our executive functioning hypothesis, we propose a re-interpretation of the most popular example of prefrontal lobe lesion and executive dysfunction, Phineas Gage. The case was first published by Harlow (1868) and since then has inspired the executive functions research and modern hypotheses, such as the somatic marker (Damasio, 1995). Not surprisingly, great attention is given to the fact that the reliable and hardworking foreman miraculously survived after a serious accident in which a tamping iron went through his frontal lobe. Even more surprisingly is that approximately 3 months after the accident, Gage was recovered enough to travel and meet his family. However, in a later report, Harlow (1868) described that previous to his injury Gage “was looked upon by those who knew him as a shrewd, smart business man, very energetic and persistent in executing all his plans of operation,” but afterward he was “no longer Gage.” Although many executive functions models have focused on this point, to illustrate our hypothesis we briefly highlight a peculiar moment that may have happened few minutes before the accident.

According to some reports, Gage’s task consisted of adding blasting powder and a fuse in a hole drilled into the rock, then using the tamping iron to pack sand into the hole above the powder. After “the powder and fuse had been adjusted in the hole, and he was in the act of ‘tamping it in,’ his attention was attracted by his men in the pit behind him” (…) “at the same instant dropping the iron upon the charge, it struck fire upon the rock, and the explosion followed” (Harlow, 1868). Within our perspective, the failure in maintaining the primary attentional focus on the task could also be described as an executive functioning failure. After a couple of months of performing the same tasks and procedures, it is possible that the individual became used to it, automating some behaviors. In the meantime, the emotional salience associated with the dangerousness of the task also decreased and the likelihood to commit a mistake gradually increased. In other words, it is possible that Gage was working in an automatic-resting state and was not able to update the new environmental demand in time. The same would have occurred with any individual working on an assembly line, or any other activity that requires repeating a series of procedures during a certain period.

On the contrary, during a lecture, teachers should be cognitively engaged to maintain the attentional focus on the topic, while delivering an informative but attractive speech. Their work require highly specialized knowledge and abilities; integrating a series of cognitive processes, such as perception, attention, memory, theory of mind, etc. The teachers in this example would be able to easily maintain low levels of stress and we could argue that they behave in a resting-control state, which is enough to keep them alert but not hypervigilant. The resting-control state is responsible for the majority of goal-oriented behaviors, and it is commonly described as the high-order cognitive abilities associated with traditional multi-component models of executive functions. Continuing with this example, after a 3-h lecture some arrogant students become bored, making noise and telling jokes. The teacher easily becomes stressed, but he/she maintains the same level of his/her lecture, while thinking about the available options to solve this annoyance. The teacher behaves in an “emotional salient-control” state, in which maintaining the executive functioning is extensively exhausting. After asking for respect and silence more than once, a laugh is heard and the teacher immediately yells at students to leave the class. The executive functioning was not enough to inhibit the response and for that reason it could illustrate an “emotional salient-automatic” behavior that solved the problem, but makes the teacher embarrassed. Without the arrogant and noisy students, the teacher relaxes and the lecture continues, now in a resting-automatic state. Although **Figure 1** suggests an adaptive process in which the organism goes through the same states, but in a specific direction (i.e., H, a, b, c), this example also shows that the executive functioning is constantly regulating the allocation of cognitive resources and emotional salience. The next time that the teacher faces a similar situation, he/she could utilize gist representations of the previous event, taking effective split-second decisions without reaching the peak of stress.

The theoretical hypothesis of executive functioning is not intended to go against any specific model or theory, but compile some important features already observed and deeply discussed by other models. Therefore, our goal is to integrate these features to suggest a more comprehensive executive functioning hypothesis, focusing on a continuous adaptive process. The similarities between different models of executive functions are evident when considering the key issues that these models seek to explain: How automatic responses are suppressed in favor of controlled responses? Are controlled responses effortful? If yes, in which way? How working memory capacity influences the suppression of automatic responses and the variability of controlled responses? What are the roles of executive functions in emotional, behavioral, and cognitive self-regulation? Considering these, some comparisons with previous models and theories could help to elucidate some features of our executive functioning perspective.

In this sense, Teuber (1972) was one of the first researchers to synthesize and discuss evidences that the PFC could anticipate consequences based upon sensory systems information, elucidating that emotional responses could be necessary to adequate cognitive functioning in some circumstances. His studies proposed an executive functions framework with a twofold gradient, including a vertical up–down gradient (related to emotional reactivity) and a horizontal back-to-front gradient (related to delay-response task) that modulates sensory systems in anticipation of future changes (Teuber, 1972). The two-fold gradient model from Teuber greatly influenced many future works, as well as the present hypothesis, because it emphasized that a certain level of emotional responses should be important to goal-oriented behaviors. Further studies from Fuster (2006), Stuss and Levine (2002), and Zelazo and Carlson (2012), for example, investigated the energizing effect of emotions in cognition and the self-regulatory aspects of executive functions.

Another important issue of our executive functioning hypothesis refers to the adaptive capacity of the organism to retrieve previous successful strategies. This idea was mostly based upon the model from Norman and Shallice (1983), which postulated that automatic responses should be suppressed in favor of more assertive ones. Initially, the idea of an automatic-controlled axis corroborates with the notion of an attentional control – described by Norman and Shallice (1983) as SAS – that emerged when routine schemas become unable to deal with non-routine circumstances. The major contribution of SAS to our perspective is based upon the idea that our attention could operate in different well-defined levels. Moreover, in accordance with the authors, the SAS indicates that individuals applied previously learned strategies to novel problems, highlighting that an executive functioning may be critical for adaptive behavior and the improvement of cognitive schemes for problem resolutions, as we discussed. By this token, it also has similarities with the theoretical and conceptual analysis of automaticity proposed by Moors and De Houwer (2006) by assuming that practice can lead to less effort in processing – something that some call automaticity – and that cognitive functioning works in a gradual manner. However, this model assumes that this dynamic process works through different features involved in automatic and controlled processes separately, and that the combination of some of these features that may influence types of behaviors/processes that we often call automatic, should in fact be considered an umbrella term for a range of features.

Interestingly, the capacity to improve previous cognitive schemes may be related to the capacity of self-monitoring and behavior inhibition since individuals might be able to anticipate future outcomes according with their own behavior (Barkley, 2001). Barkley (2001) also suggests that an executive functions model may take into account an evolutionary principle of gradualism. Therefore, executive functions may be understood as a form of mental capacity that might be observed in other species, in which human beings show the higher capacity of mental representation concerning future perspectives and the ability to inhibit undesirable behaviors (Barkley, 2001). In this sense, the capacity to constantly update environmental contingencies and inhibit behaviors also seems to be a central aspect in our perspective since it allows the individual to transit by all behavioral states (**Figure 1**), predicting hypothetical futures based upon previous experiences and them inhibiting an automatic response in favor of a controlled one (SAS influence).

More recently, Asp et al. (2013) defined this monitoring/updating characteristic of inhibitory control as the False Tag Theory (FTT), suggesting that affective processes signalize inappropriate responses (“false tags”). In order to include affective processing, the authors also suggest that the capacity to “false tag” inappropriate responses has a limited resource, which can be taxed during periods of high cognitive work (Asp et al., 2013). In this sense, the FTT extended the Somatic Marker Theory (SMT) proposed by Damasio (1996), which elucidated that decision-making is an emotion-dependent process that is wrought through the repetition of experiences. However, FTT proposes how affective signaling could operate with cognitive processes, showing that if there is a concurrent requirement of both “false tagging” to perceptual and cognitive representations, there can be competition for the “false tagging” resource and the efficacy of each process may be decreased (Asp et al., 2013). For our perspective, it corroborates with the idea that cognitive processes are influenced by emotional salience, facilitating or biasing cognitive processes.

Finally, our executive functioning hypothesis is in accordance with the Hot-Cold Decision Triangle (Yang et al., 2012) and the Tri-dimensional Processing model (Varga and Hamburger, 2014). First, both models emerged as a criticism of standard dual-processing models, each one focusing on different arguments. Yang et al. (2012) discuss that effortlessly and effortful cognitive engagement are mediated by an emotional processing, the model suggests that optimal and healthier decisions are influenced by the extent to which System 1 overlaps System 2. Our perspective of executive functioning is different from the Hot-Cold Decision Triangle because their model assumes a prescriptive framework in which optimal decisions are made with less emotional engagement. Moreover, the Tri-dimensional Processing model also added an important issue concerning the continuous dimensions of information processing. However, the authors did not suggest it as an executive functioning outcome, derived from the interaction between automatic-controlled behaviors and emotional salience. This interaction was deeply discussed by Ernst (2014), who suggested that the efficient adaptive behavior would result from the balance between appetitive (striatum-dependent) and avoidant (amygdala-dependent) processes. In his model, PFC works as a “conductor” and regulates approach-avoidant behaviors according to environmental demands (Ernst, 2014).

## Future Perspectives

Instead of presenting a new model, the executive functioning hypothesis presented here should question the current hierarchical and categorical framework and be interpreted as an alternative perspective for future studies. An executive functioning perspective may favor novel discussions about which individual factors can mediate the balance between emotional salience and automatic-controlled behaviors. For example, the threshold of the amount of stress that someone could tolerate to maintain goal-oriented behaviors (emotional salient-controlled state) and not act impulsively (emotional salient-automatic state), may be influenced by personality traits and early experiences, as well as developmental stages and genetic background. Specifically, positive and negative urgency are individual characteristics that are marked by the ineffective control of decisions under extreme emotional states, and particularly, people react differently under different emotional states (Cyders and Smith, 2008). There are some individuals who react rashly to emotional states with positive valences (which means they present positive urgency), and there are people who can react rashly only under negative emotional states (which means they present negative urgency). It has been documented that a combination of personality factors contributes to positive and negative urgency (Gay et al., 2008). With regards to genetic factors, there are evidences suggesting that polymorphisms in the dopamine receptors and serotonin transporters genes are related to positive and negative urgency related to decision-making [for a review about positive and negative urgency and those consequences for behavior, see (Billieux et al., 2010).

Moreover, as reviewed by Peterson and Welsh (2014), an additional feature that should be clearly elucidated concerns the emotional-salience axis. Although dual-processing models theoretically assume that “cold” and “hot” are not independent features of executive functions, in practice the “thermal gradient” between them is still poorly understood. Future researches in experimental psychological should manipulate the “temperature” of a single task (Peterson and Welsh, 2014). Using an interesting approach combining behavioral economic and computational modeling, Summerfield et al. (2011) investigated the allocation of higher-order cognitive strategies by manipulating the volatility of the environment (i.e., the level of uncertainty when environment can change rapidly and without warning). On the other hand, a computational working memory model seems to fit the individual’s behavior better during high-volatile (i.e., uncertainty) scenarios. Interesting, the authors suggested that the optimal (Bayesian) decision model predicted-related activity in more posterior regions of the medial PFC while anterior regions of the medial PFC actively respond when decisions are based on motivational information (Summerfield et al., 2011). Likewise, our recent work suggested that during scenarios in which participants have no knowledge about their own performance and lower emotional arousal responses (for more details see Huang et al., 2013), their behavior is mostly modulated by the use of the available information in the decision scenario. Conversely, if some feedback is provided, participants tend to use less or even no available information about the environmental risks (Kluwe-Schiavon et al., 2016). These data are in line with the idea that contingency learning, habitual behavior, and goal-directed behavior could be studied as a continuum modulated by the uncertainty of the environment. Furthermore, manipulating these variables – environmental volatility or the presence of feedback – in experimental contexts might be an effective way to understand behavioral and neural mechanisms behind updating previously learned schemas. Bearing in mind that our model considers an executive functioning that updates itself continuously, integrating both external and internal homeostatic changes, stress paradigms, such as the Trier Social Stress Test (TSST), seem to be a promising option. In this sense, it should be noted that a recent study showed that stressful situations mimicked by the TSST, elicited a stronger activation of the PFC associated with a risky decision-making process (Gathmann et al., 2014).

Additionally, one could question how our executive functioning hypothesis would account for dissociation between different components of executive functions that can be observed in health and mental illness (e.g., how would this hypothesis account for impaired reasoning and problem solving but intact working memory, or impaired working memory but intact inhibitory control). As supported by Summerfield et al. (2011), a dimensional perspective of executive functioning would be able to identify the amount of environmental volatility that someone may confront until goal-oriented responses shift to habitual responses. In this sense, it is still possible that someone could have an intact working memory and impaired problem solving, especially because according to our hypothesis, good performance in working memory could favor the individual to respond to the environmental demand based upon ongoing feedback, without necessarily allocating excessive cognitive resources to planning and problem solving. The same could occur with someone who has an impaired working memory but intact inhibitory control. In this case, someone that presents a decrease in working memory capacity (meaning that less information is retained in his/her memory span) could also present high behavioral variability, which is different from impulsivity or impairments in inhibitory control. It is possible that the same person would not have problems in inhibiting an automatic response when a high emotional-salient stimulus is shown. However, it is true that according to our hypothesis, it would be harder to explain someone who has an impaired working memory but has no difficulties in successfully planning goal-oriented behaviors. This point is important, once more, to illustrate that the current categorical multi-component view of executive functions is still focused on classifying which behaviors should represent cognitive flexibility or inhibitory control (Diamond, 2013), instead of understanding each behavior as a reflex of an adaptive process.

Finally, when we propose different axis we are not assuming consistently that there is a marked frontier between each axis, but we perform an estimation that is relative for understanding proposes. This criteria is similar to that suggested by Moors and De Houwer (2006) when they flagged one limitation of theories that use gradual models. In this sense, further studies could be done to clearly define emotional-salience states and automatic-controlled responses in order to measure it accurately. Based upon a behavioral perspective, it is possible to infer that automatic responses could be defined as those reflexive or conditioned responses. We hypothesized that automatic responses are more likely to occur in an environment with a very high or very low emotional-salience. Moors and De Houwer (2006) discussed that we can identify automaticity by viewing components/features that probably play a more significant role in less controlled processes (e.g., unconscious, unintentionally, or autonomous processing). This view indicated the need to use decompositional methods to investigate automaticity and highlighted that some combinations of different features can give us insight into the dynamics of cognitive control (Moors and De Houwer, 2006). On the other side of the automatic-controlled axis, controlled responses could be defined as those goal-oriented, non-conditioned responses that according to our hypothesis, are more likely to occur with middle-level emotional-salience. However, a clear definition of emotional-salience state is needed. For example, as discussed by Shields et al. (2016) and extensively described by McEwen et al. (2015), the effects of stress on executive functions goes beyond cortisol alone and the HPA-axis, since circulating proinflammatory cytokines can also have an impact on working memory (Marsland et al., 2006) and cognitive flexibility (Levandowski et al., 2016). Further studies should investigate multiple hormones and immune system processes in order to deeply understand the biological mechanisms behind the effects of stress on executive functions (Shields et al., 2016).

## Author Contributions

BK-S substantially contributed to the conception of the model and design of the work; AND, drafting the work and revising it critically for important intellectual content; AND final approval of the version to be published; AND agreement to be accountable for all aspects of the work in ensuring that questions related to the accuracy or integrity of any part of the work are appropriately discussed and resolved. TWV contributed to drafting the work and revising it critically for important intellectual content; AND final approval of the version to be published. BS-V substantially contributed to the conception of the model and design of the work; AND drafting the work. LM-D final approval of the version to be published; AND agreement to be accountable for all aspects of the work in ensuring that questions related to the accuracy or integrity of any part of the work are appropriately discussed and resolved. RG-O substantially contributed to the conception of the model and design of the work, AND final approval of the version to be published; AND agreement to be accountable for all aspects of the work in ensuring that questions related to the accuracy or integrity of any part of the work are appropriately discussed and resolved.

## Conflict of Interest Statement

The authors declare that the research was conducted in the absence of any commercial or financial relationships that could be construed as a potential conflict of interest.
